# Surgical Navigation, Augmented Reality, and 3D Printing for Hard Palate Adenoid Cystic Carcinoma En-Bloc Resection: Case Report and Literature Review

**DOI:** 10.3389/fonc.2021.741191

**Published:** 2022-01-04

**Authors:** Mónica García-Sevilla, Rafael Moreta-Martinez, David García-Mato, Gema Arenas de Frutos, Santiago Ochandiano, Carlos Navarro-Cuéllar, Guillermo Sanjuán de Moreta, Javier Pascau

**Affiliations:** ^1^ Departamento de Bioingeniería e Ingeniería Aeroespacial, Universidad Carlos III de Madrid, Madrid, Spain; ^2^ Instituto de Investigación Sanitaria Gregorio Marañón, Madrid, Spain; ^3^ Servicio de Cirugía Oral y Maxilofacial, Hospital General Universitario Gregorio Marañón, Madrid, Spain; ^4^ Servicio de Otorrinolaringología, Hospital General Universitario Gregorio Marañón, Madrid, Spain

**Keywords:** surgical navigation, augmented reality, 3D printing, head and neck cancer, adenoid cystic carcinoma

## Abstract

Adenoid Cystic Carcinoma is a rare and aggressive tumor representing less than 1% of head and neck cancers. This malignancy often arises from the minor salivary glands, being the palate its most common location. Surgical en-bloc resection with clear margins is the primary treatment. However, this location presents a limited line of sight and a high risk of injuries, making the surgical procedure challenging. In this context, technologies such as intraoperative navigation can become an effective tool, reducing morbidity and improving the safety and accuracy of the procedure. Although their use is extended in fields such as neurosurgery, their application in maxillofacial surgery has not been widely evidenced. One reason is the need to rigidly fixate a navigation reference to the patient, which often entails an invasive setup. In this work, we studied three alternative and less invasive setups using optical tracking, 3D printing and augmented reality. We evaluated their precision in a patient-specific phantom, obtaining errors below 1 mm. The optimum setup was finally applied in a clinical case, where the navigation software was used to guide the tumor resection. Points were collected along the surgical margins after resection and compared with the real ones identified in the postoperative CT. Distances of less than 2 mm were obtained in 90% of the samples. Moreover, the navigation provided confidence to the surgeons, who could then undertake a less invasive and more conservative approach. The postoperative CT scans showed adequate resection margins and confirmed that the patient is free of disease after two years of follow-up.

## Introduction

Salivary gland tumors account for approximately 5% of head and neck cancers (1) and are, in most cases, benign. Only 20% of these neoplasms are malignant, although this rate varies depending on the gland of origin (2, 3). Unlike most head and neck cancers, which are squamous cell carcinomas, salivary gland tumors comprise multiple histologic entities, each presenting a different clinical behavior (4). Mucoepidermoid carcinoma is the most frequent malignancy, followed by adenoid cystic carcinomas, representing less than 1% of all malignancies in head and neck cancers (5). These tumors can appear in both minor and major salivary glands. Most major salivary tumors occur in the parotid glands, while the palate is the most common location for minor salivary gland tumors (6).

Tumors located in the palate are often diagnosed in advanced stages of the disease due to indolent growth during early stages, with vague and unspecific symptoms (5, 7). In some cases, this phenomenon leads to extensive involvement of surrounding structures, such as the nose, paranasal sinuses, orbits, and even the middle cranial fossa, with the subsequent implication of vital structures such as the internal carotid artery, the jugular vein, and cranial nerves.

Regardless of whether it is followed by radiotherapy, surgery is the treatment of choice for midface tumors. To date, the surgical management of these tumors consists of radical maxillectomies combined with transfacial approaches, which are usually associated with significant functional and aesthetic sequelae. However, achieving safety margins is a therapeutic challenge. This is due to the complexity of the anatomical region, the reduced field of vision, the restrictive surgical field hindering the access and maneuverability of surgical instruments, and the risk of complications (bleeding, nerve injuries, or even cerebrospinal fluid leaks) (8). In addition, middle third tumors are frequently irregular in shape and invade neighboring structures. Consequently, it is not uncommon to set suboptimal cutting trajectories, which results in a high rate of positive margins.

An alternative approach for centrofacial tumors is the endoscopic-based resection, which considerably reduces the morbidity of surgery compared to open craniofacial resections. However, open surgery is still necessary for the most advanced stage cancers, often combined with endoscopic resection (9).

In these scenarios, tools facilitating local control during surgical resection and confirming adequate margins while minimizing morbidity are capital (10).

### Computer-Assisted Surgery in Oral and Maxillofacial Surgery

In recent years, intraoperative navigation in craniofacial surgery has become an effective tool, improving results and safety while minimizing the risk of injuries (11, 12). Surgical navigation, also called image-guided surgery, was first described in the medical literature in the 70s when framed stereotaxy was introduced. This involved rigid fixation of the region to be treated, a situation that, on many occasions, entails significant mechanical limitations for the surgeon. Navigation was initially confined to neurosurgery as it provides a rigid and stable frame (13). However, technical advances in image processing and computed tomography allowed the development of frameless stereotaxy devices over the years, enabling the location of tools in 3D space intraoperatively without the need for rigid fixation. This 3D information, synchronized with preoperative computed tomography (CT) or magnetic resonance images (MRI), has given rise to 3D intraoperative navigation systems (14).

Surgical navigation enables the translation of the preoperative plan to the operating room, where anatomical structures can be identified in real-time. This technology allows intraoperative orientation regarding resection margins and surrounding vital structures, improving the safety and accuracy of the procedure. Also, recent advances have enabled navigation systems to be used not only as a simple localization device but also as a measurement tool that can provide other relevant information to the surgeons during different stages of the procedure.

There are currently several navigation solutions to track the positions of surgical instruments relative to patient anatomy. Usually, the selection of the tracking device depends on the specific surgical procedure. Optical tracking systems based on infrared cameras are the most common solution in interventions where a direct line of sight between the camera and the surgical instruments can be maintained. They are widely chosen, as they provide a large field of view and high accuracy (13, 15). In these interventions, retroreflective markers are fixed to the patient through a dynamic reference frame, allowing tracking of the patient’s movements. The real-time position of these sensors is captured by the camera and translated to the navigation software. Other navigation solutions include mechanical or electromagnetic tracking systems (16).

Recent studies have also presented augmented reality as a tool for navigation. With this technique, virtual models can be superimposed onto the patient’s anatomy instead of displaying them on an external screen. The virtual components can be represented in place either by following a manual alignment with the patient’s anatomy (17, 18) or by using different tracking solutions such as electromagnetic or optical tracking systems (19, 20), features recognition (21), or optical markers (22–25). The information can be presented on an external screen, a smartphone, or a head-mounted display such as the HoloLens. Some examples include spine surgery (26, 27), craniosynostosis treatment (24), orthopedics (23, 28), or dental implant placement (29).

One of the most critical steps in surgical navigation is image-to-patient registration. This technique estimates the transform that aligns the preoperative images with the patient during the intervention. Registration can be either rigid or nonrigid. Nonrigid registrations are used as a secondary registration for pose refinement or to work with nonrigid surgical fields (30, 31). The most common algorithm is paired-points registration, where corresponding anatomical or artificial landmarks are located in the image and patient. Many systems include a secondary registration for refinement based on surface-points matching. In oral and maxillofacial surgery, the selected paired points are either anatomical landmarks or artificial fiducials, including skin stickers (32), dental splints (33), or bone-implanted screws (34). Surface-points matching is also widely applied by collecting points in the facial skin surface and aligning them with their corresponding landmarks in soft tissue models obtained from preoperative images (32, 35, 36).

The progressive evolution of intraoperative navigation systems has led to the use of this technology almost routinely in the field of neurosurgery (skull-base surgery, vascular lesions), otorhinolaryngology (endoscopic sinus surgery, lateral skull-base surgery, cerebrospinal fluid leaks), and, recently, orthopedic surgery (hip and knee arthroplasty, spinal procedures) (13). However, the extended application of this technology has not been widely evidenced in maxillofacial surgery (37, 38). In 2015, Dubois et al. (39, 40) presented the benefits of surgical navigation for accurate implant positioning secondary to orbital trauma by performing a cadaveric study. Four years later, Wu et al. (41) demonstrated the feasibility of using surgical navigation for zygomatic implant placement. Many other studies found in the literature have proved the benefits of surgical navigation, especially for complex procedures where a personalized approach is required.

Some applications of computer-assisted surgery (CAS) and intraoperative navigation have been described in this field, including orbital reconstruction (42, 43), implant surgery (44–46), or orthognathic surgery (47). Surgical navigation has also been used in tumor removal to delineate surgical margins and achieve safe and accurate resections (11, 48–50). The results are promising, but the clinical adoption is still reduced. The main limitations of this approach include complexity, technical support, cost, steep learning curve, or the rigid fixation of navigation references (14, 49), which are perceived as an entry barrier. Nevertheless, several commercial solutions exist in the market for oral and maxillofacial surgery. Some examples of navigation systems based on optical tracking are Columbia Scientific SIM/Plant software (Columbia scientific, USA) (51, 52), VISIT surgical navigation software (Vienna, Austria) (53, 54), and Vector Vision (Brainlab, Munich, Germany) (45) for dental implants placement, or Stryker Leibinger navigation system (Stryker, Leibinger, Freiburg, Germany) (50, 55, 56) for the removal of foreign bodies and tumors, or repairing orbital bone fractures. Although some studies have presented AR-based surgical navigation systems for oral and maxillofacial surgery applications (24, 57, 58), no commercial systems are available.

The application of CAS and intraoperative navigation in the midface, specifically in resections involving the maxilla and the middle cranial fossa, can become a valuable tool in complex procedures. Additionally, the presence of bone structures that do not modify their contours and volumes during the intraoperative process due to surgical maneuvers is an essential advantage for navigation (59). Therefore, these interventions provide an adequate scenario for navigation and can highly benefit from this technique.

In addition to intraoperative navigation, there are more resources that CAS procedures can provide in this field. Advances in preoperative planning with computer-aided design (CAD) and computer-aided manufacturing (CAM) have enabled the development of customized and prefabricated templates that facilitate both surgical resections and reconstruction (60–62). The recent introduction of intraoperative imaging, particularly computerized tomography (CT), has solved the limitations of morphological change, intraoperative edema, and soft tissue distortion that appear during the surgical resection and could not be considered in preoperative planning. Thus, the acquisition of intraoperative images once the resection surgery has been carried out provides additional information, allowing the verification and the achievement of adequate surgical margins (63, 64).

With the same objective of safety, accuracy, and minimization of sequelae in the treatment of tumors that involve the maxilla and midface, it is worth mentioning the endoscopically-assisted maxillectomy. The endoscopic transnasal approach provides advantages such as better control of the medial and posterior margins and the possibility of avoiding transfacial approaches when combined with intraoral procedures, minimizing the radicality of surgical resections (10).

### Adenoid Cystic Carcinoma

Adenoid Cystic Carcinoma (ACC) is a rare malignant tumor accounting for 1% of head and neck cancers and 10% of salivary gland tumors (65). It is commonly found in palate small salivary glands, from where it spreads slowly but aggressively. These tumors settle in the upper palate and maxillary region and tend to local infiltration and perineural spread. Consequently, they may behave in an indolent and silent manner until late diagnosis, appearing as destructive masses that can even involve intracranial structures. In addition, this histologic type is characterized by a high predisposition to systemic dissemination, mainly hematogenous (lung, liver, brain, and bone) and lymph nodes. They present a high propensity to local recurrence and distant metastasis (5). The primary treatment consists of surgical removal with clear margins and complementary radiotherapy when needed. Data on the efficacy of systemic therapy or radiotherapy in recurrent or metastatic salivary gland tumors are limited, with some benefits described in proton-based radiotherapy (66–68) or the recent systemic use of Lenvatinib (69). Consequently, an effective primary surgical treatment with adequate margins is the best prognostic factor for these patients.

However, this intervention is highly challenging due to the occasional centrofacial tumor location, complex elective surgical approach, limited line of sight, and the need for immediate reconstruction. Additionally, the tumor boundaries are difficult to discriminate from the normal surrounding tissue.

In this type of intervention, where complex anatomy is present and high accuracy is needed, surgical navigation becomes a valuable tool to improve clinical outcomes. It can provide guidance that can help achieve accurate safety margins and protect vital structures. However, despite its great potential in these clinical applications, there are currently limited studies using CAS for midfacial tumor resection. Wei et al. (11) tested surgical navigation in patients who underwent surgery near the skull base, including five patients with adenoid cystic carcinoma at minor salivary glands of the palate. Their approach was limited by the invasive attachment of a reference frame to the patients’ forehead and an image-to-patient registration based on non-precise anatomical landmarks, which can significantly reduce the navigation accuracy. Tarsitano et al. (49) followed a similar setup for maxillary tumors resection, screwing a dynamic reference frame to the patient’s skull.

The aim of this study is to present and assess the accuracy of three different alternatives for surgical navigation in head and neck tumors based on 3D printing, optical tracking, and augmented reality visualization. These alternatives are less invasive than previous solutions (11, 49) and more convenient for these procedures than conventional registration solutions used in other disciplines such as neurosurgery as they do not involve fixation of the patient’s head. A 3D printed patient-specific phantom was used for validation and assessment of the three navigation systems. One of the proposed solutions was then used to guide the tumor resection of a patient presenting a central palate carcinoma invading the nasal fossa floor and septum.

## Methods

### Clinical Case

**Figure 1:**
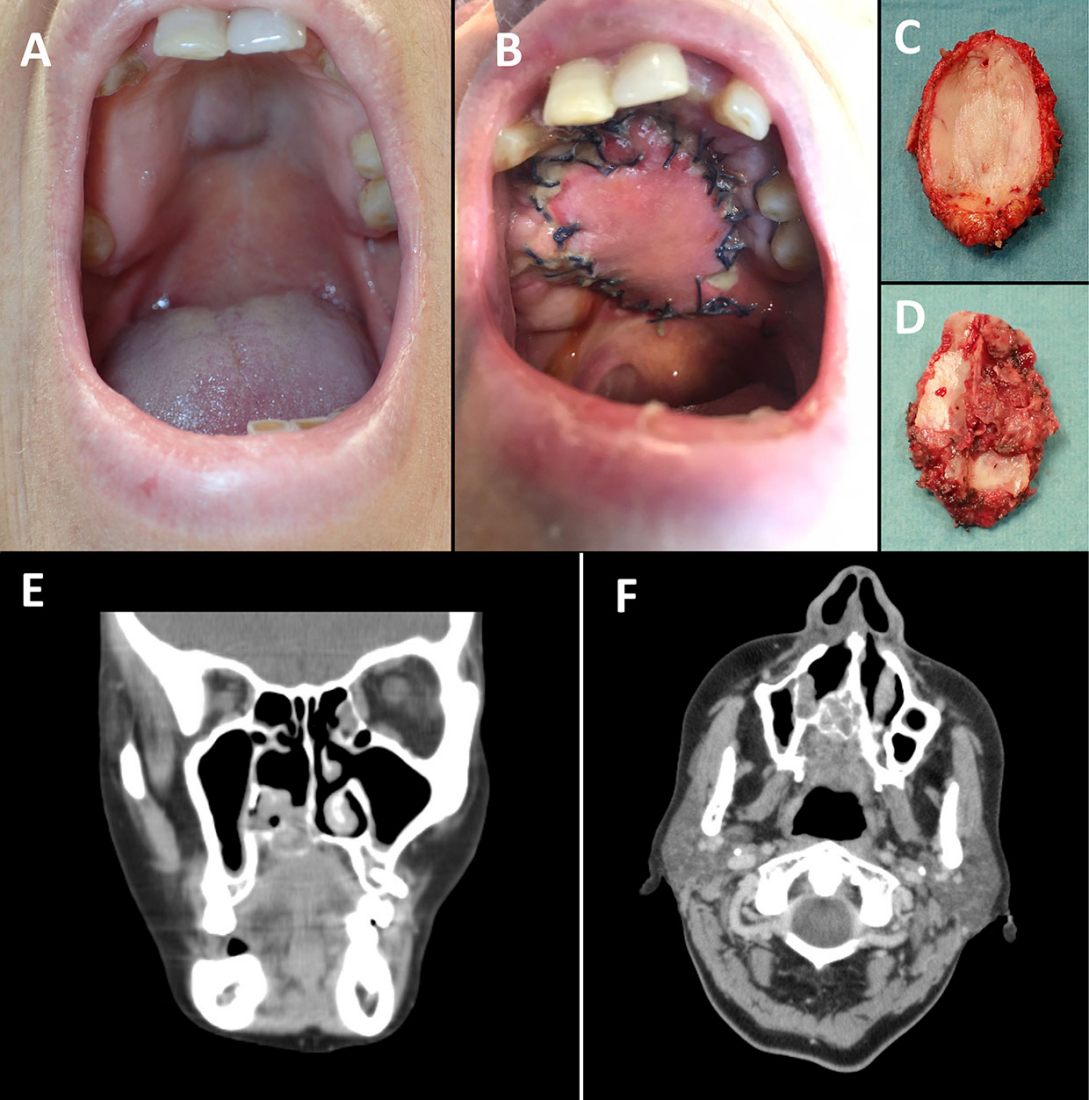
Hard palate midline submucous bulging lesion **(A)** and palate reconstruction with radial forearm free flap (3 weeks after surgery) **(B)**. Resected specimen from **(C)** palate and **(D)** nasal view. **(E)** Coronal and **(F)** axial views of the CT image.

A 62-year-old woman was referred to the Oral and Maxillofacial Unit at our center for treatment. The patient presented an exophytic tumor of approximately 3x2 centimeters in the middle of the hard palate with normal oral mucosa (**Figure 1A**). Endoscopy showed a nasal extension of the lesion, and biopsy results confirmed an adenoid cystic carcinoma. A CT scan showed hard palate bony erosion, invasion for the nasal septum and floor of the right fossa, and an intact ipsilateral inferior turbinate (**Figures 1E, F**).

The chosen procedure consisted of an endoscopic nasal approach, a navigated transoral resection of the central palate with at least 2 cm margin (**Figures 1C, D**), and immediate reconstruction of the central hard and soft palate with a radial forearm free flap (**Figure 1B**). Alternative surgical approaches considered were a Le Fort I osteotomy (downfracture of the whole maxilla and resection of the central part) or a IIb maxillectomy (Brown classification, sacrificing the intact alveolar process and denture) reconstructed with a fibula free flap. Our purpose was to achieve functional rehabilitation, including a tight palate seal and maintaining the whole alveolar process of the maxilla. The proposed solution presented a more straightforward reconstruction involving only soft tissue, as bone reconstruction is not needed in horizontal class a defects.

The preoperative CT scan was used to extract the 3D anatomical models (bone and tumor) and perform the preoperative plan, defining the desired tumor margins for the resection. Using Autodesk Meshmixer software (Autodesk, Inc., USA), we increased the size of the tumor model 1 cm. The intersection of this model with the bone in the palate determined the surgical margins (1 cm margin with a thickness of 1 mm). Before image acquisition, five screws were attached to the maxilla above the upper teeth under local anesthesia as proposed by Zavattero et al. (70). This procedure provides unobtrusive, rigid, and exact landmarks that are clearly visible on virtual data sets (CT images) as well as during the navigation procedure. The position of these screws was identified in the scan for later use during intraoperative image-to-patient registration.

### Surgical Navigation: Simulation

**Figure 2:**
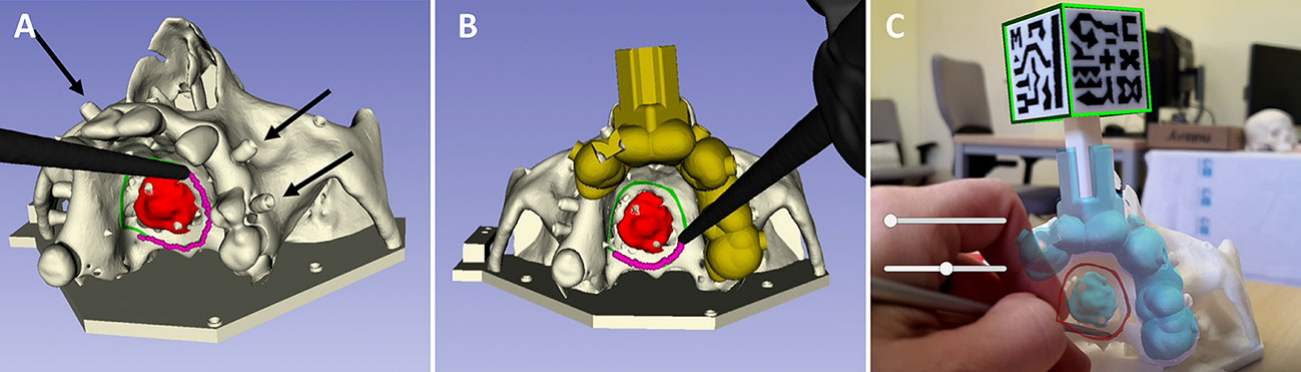
Solutions for surgical navigation tested on the patient’s anatomical model: **(A)** navigation with an optical tracking system and registration with screws (black arrows); **(B)** navigation with an optical tracking system and registration with a splint; **(C)** navigation with an AR application and registration with a cubic marker.

Based on the anatomical models of the patient, we designed and manufactured a phantom in polylactic acid using the desktop 3D printer Ultimaker 3 extended (Ultimaker B.V., NL). The phantom was used to simulate the intervention and to test the precision of three different solutions for surgical guidance (**Figure 2**).

The first solution consists in using an optical tracking system (NDI Polaris Spectra, CA) for computer-assisted navigation (**Figure 2A**). We attached a 3D printed dynamic reference frame to a silicone jig fabricated to fit on the patient’s upper left teeth. The dynamic reference frame is used to compensate for head movements. This silicone jig consists of a mass given the shape of the patient’s teeth during its malleable state. A picture of the fabrication procedure is provided as **Supplementary Material**. The image-to-patient registration is performed using the screws described in the previous section as artificial landmarks.

A second solution involves the same tracking device, but the reference frame is installed by means of a splint (**Figure 2B**) instead of using a silicone jig. This splint is designed from the 3D models of the teeth obtained from the preoperative images, and 3D printed in a biocompatible resin (Biomed Clear), using the Formlabs Form2 (Formlabs Inc., Somerville, MA, USA) 3D printer. In contrast to the first method, the registration in this case is computed with artificial landmarks added on the splint during the design process.

Finally, the third solution uses AR for surgical navigation (**Figure 2C**). We developed a specific smartphone app to visualize the patient’s anatomy and the tumor margins. The application was implemented on the Unity platform (version 2019.3), using the Vuforia development kit (Parametric Technology Corporation Inc., Boston, MA, USA) for pattern recognition. The application displays the 3D models of the bone, tumor and surgical margins (obtained from the preoperative image and planning) and includes buttons to change the visibility of the models (modify opacity or hide). In order to display the virtual models in the correct position with respect to the patient’s phantom, we attached a 3D-printed marker to the splint in a fixed and known position. This AR marker contains a unique black and white pattern printed using the double extruder functionality of the Ultimaker 3 extended 3D printer. The smartphone’s camera detects this marker and displays the virtual models on the screen on top of the patient. **Figure 2C** and the video included as **Supplementary Material** show the appearance of the developed AR application.

The three configurations were tested on the phantom, where we performed the corresponding registration procedure for each configuration. After that, each navigation system displayed the position of the surgical margins defined preoperatively. Using the optical tracker and the pointer, we collected points (with distances of 1 mm between each other) following the indicated surgical margins. The process was repeated three times for each configuration, including the registration step. A similar number of points was recorded for each sample (around 100 points).

Inaccuracies in the registration step would generate errors in the displayed margins. Therefore, the collected points would present deviations from the position of the real surgical margins. In order to assess the accuracy for each configuration, we computed the distances between the collected points (following the margins indicated by each navigation system) and the real position of the tumor margins. For that, we obtained the closest point of the resection margins (a 3D model or pointcloud) to every recorded point and stored the distance. Then, we computed the median and quartiles for each solution. Finally, we conducted a statistical analysis to identify significant differences in accuracy between methods.

The surgical margins used as ground truth for evaluating the three methodologies were the ones defined during preoperative planning with the preoperative CT (1 cm margin). The real position of the tumor margins was obtained during assessment thanks to the rigid attachment of a reference frame to the phantom and a registration performed with artificial landmarks distributed all over the surface to ensure accurate registration. These artificial landmarks were added as conical holes in the bone region and the base of the phantom and can be seen in **Figure 2**.

### Surgical Navigation: Setup

**Figure 3:**
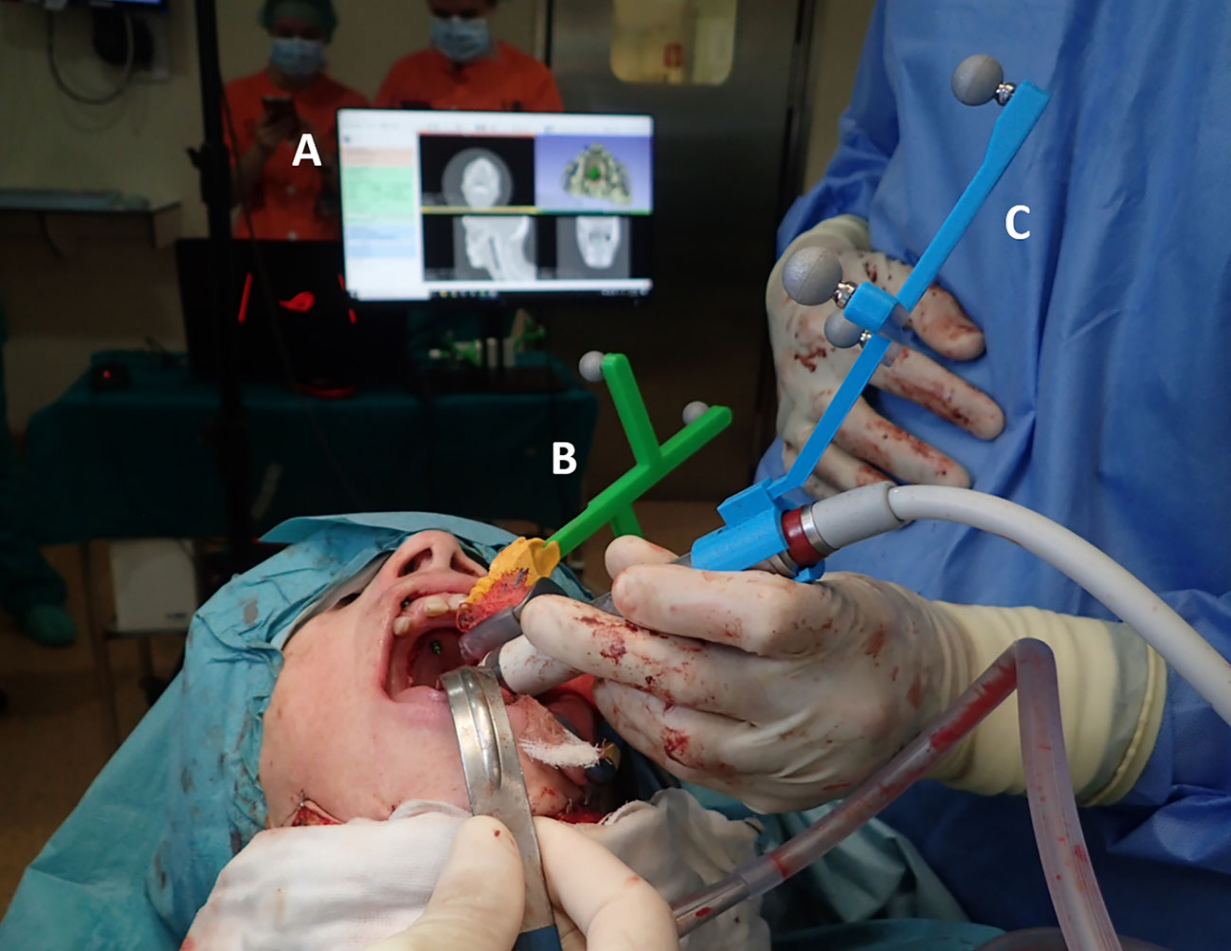
Surgical navigation setup during the intervention: **(A)** surgical navigation software; **(B)** 3D-printed patient’s dynamic reference frame; **(C)** 3D-printed adaptor for tracking of the piezoelectric handpiece.

Apart from tracking the patient with the 3D-printed dynamic reference frame (**Figure 3B**), two different instruments were tracked during surgery: a pointer tool to record points and a piezoelectric handpiece for tumor resection. We designed and 3D printed an adaptor with optical markers to fit in the handle (**Figure 3C**) for handpiece tracking. An additional tool was also designed and 3D-printed to fit the instrument at a specific position. This tool included six small conical holes for registration. The **Supplementary Material** shows a picture of the designed tool. Finally, as the handpiece is composed of an interchangeable saw with a non-fixed rotation around the longitudinal axis, we added an extra step in the registration procedure. This step consists in recording the position of the saw tip and automatically finding the rotation that corrects the orientation of the saw in the navigation scene. That is the rotation that minimizes the distance between the virtual tip point and the recorded point. The pointer and all 3D printed tools, including the dynamic reference frame, were sterilized before the intervention to maintain the asepsis of the surgical field.

We developed a custom module for surgical navigation in the 3D Slicer platform (71), a free and open-source software package for clinical and biomedical applications. We used the SlicerIGT kit (72) and the PLUS toolkit (73) to define the graphical user interface and manage the transforms sent by the optical tracker through the OpenIGTLink protocol. Intraoperative imaging was not used during the procedure. However, our software allowed the visualization of the CT preoperative image and the 3D models obtained of the patient (bone, tumor, and surgical margins) and the position of the instruments (**Figure 3A**). The three views of the CT (axial, sagittal, and coronal) could be updated in real-time to match the position of the instrument’s tip for better guidance. The software also included other functionalities, such as modifying the point of view in the 3D view or recording points. The **Supplementary Material** includes a video showing the use of the software during the intervention.

### Surgical Navigation: Intervention

**Figure 4:**
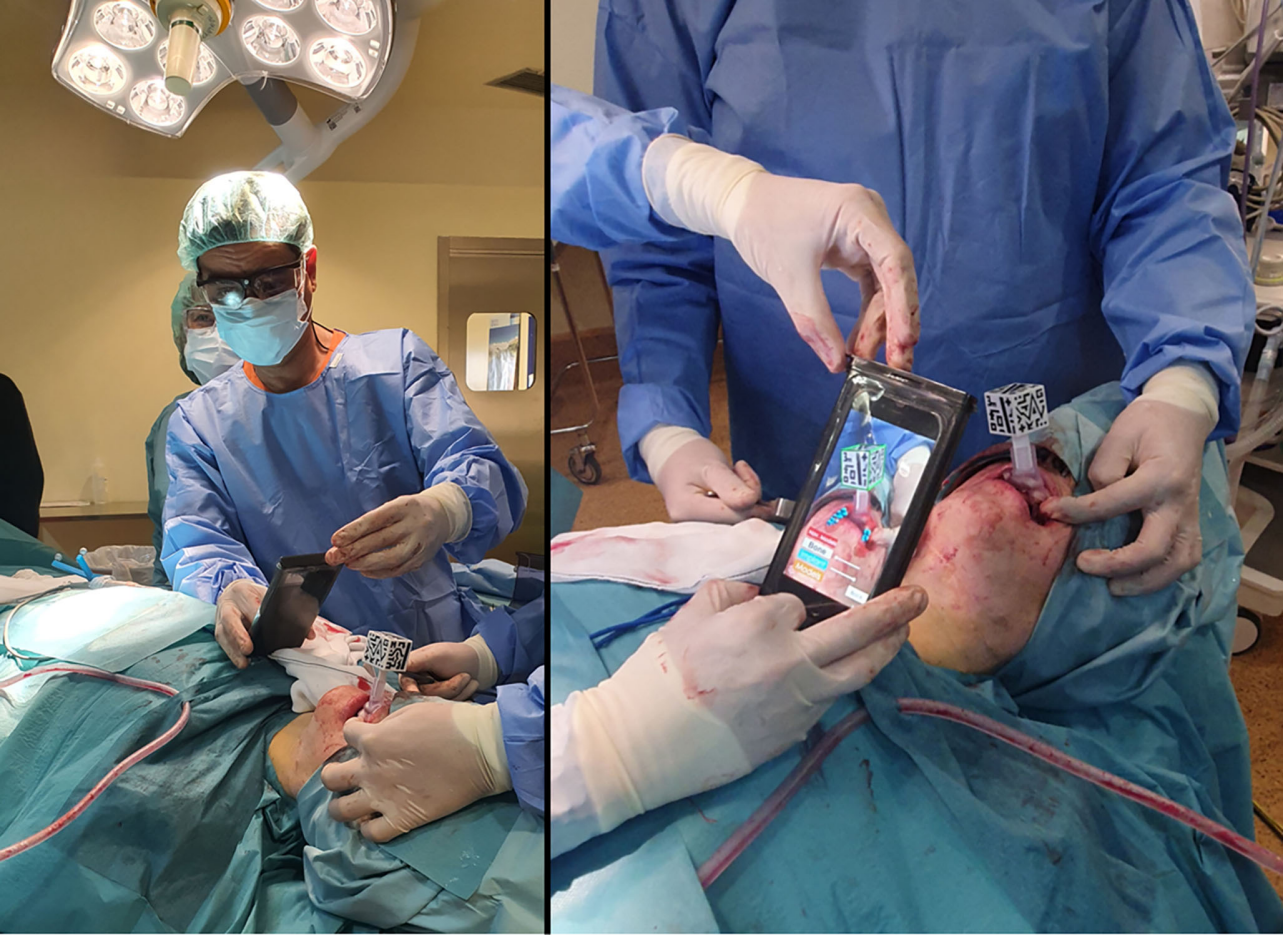
Use of the augmented reality app during the intervention.

Resection margins were controlled in real-time using the developed software through constant visual feedback displayed on a screen adjacent to the surgical field. During the intervention, we increased the surgical margin 1 cm from the preoperative segmentation to ensure adequate en-bloc resection with 2 cm of tissue free of disease. The final resection margins were recorded using the pointer. While not used for surgical guidance, AR was tested on the patient to validate the AR setup using the splint (**Figure 4**). The smartphone was introduced in a sterile case (CleanCase, Steridev Inc., Lansing, MI, USA) so that surgeons could hold it close to the patient.

After resection, a radial forearm free flap was harvested and placed to reconstruct the palate with an adequate seal. A postoperative CT scan was performed a week after surgery to assess the surgical outcome. Navigation accuracy was measured as the absolute distance between the points recorded intraoperatively and the real resection margins identified in the postoperative CT. A secondary CT scan was acquired 15 months after surgery for the patient’s follow-up.

## Results

### Surgical Navigation: Simulation

We analyzed the accuracy provided by each navigation solution on the 3D printed phantom by computing the distances between the collected points during guidance and the real resection margins. The results for each configuration are presented in **Table 1**.

**Table 1:** Mean and standard deviation of the distances between the tumor margins and the collected points with each navigation solution.

Navigation solution	Median	Q1	Q3
OTS (registration with screws)	0.57	0.34	0.81
OTS (registration with surgical guide)	0.61	0.30	0.98
AR	0.40	0.14	1.29

OTS, optical tracking system; AR, augmented reality; Q1, Q3, first and third quartiles (25^th^ and 75^th^ percentile).

All methods presented median values below 0.7 mm. Most of the points recorded using the optical tracking system for guidance presented deviations from the surgical margins below 1 mm. The results obtained when using AR for guidance presented the lowest median value (0.4 mm). However, they also presented the highest variation, with an interquartile range (IQR) of 0.89 mm compared to the ones obtained with optical tracking, where the IQRs for the screws and surgical guide configurations were 0.24 mm and 0.37 mm, respectively.

A Kruskal-Wallis test was performed to explore the differences between each configuration proposed for this study. No statistically significant differences were obtained [H(3) = 4.27, p = .12]. Therefore, we can conclude that the three configurations present similar accuracy. The configuration using screws for registration was the one presenting lower error. Thus, it was the one chosen for the intervention.

### Surgical Navigation: Intervention

The selected navigation system (optical tracker with screws for registration) was successfully used for guidance during the resection. Surgical instruments were accurately tracked with respect to the patient’s anatomy, providing valuable feedback to the surgeons. The registration step was repeated three times during the intervention, obtaining a fiducial registration error of 0.77, 0.93, and 0.81 mm.

**Figure 5:**
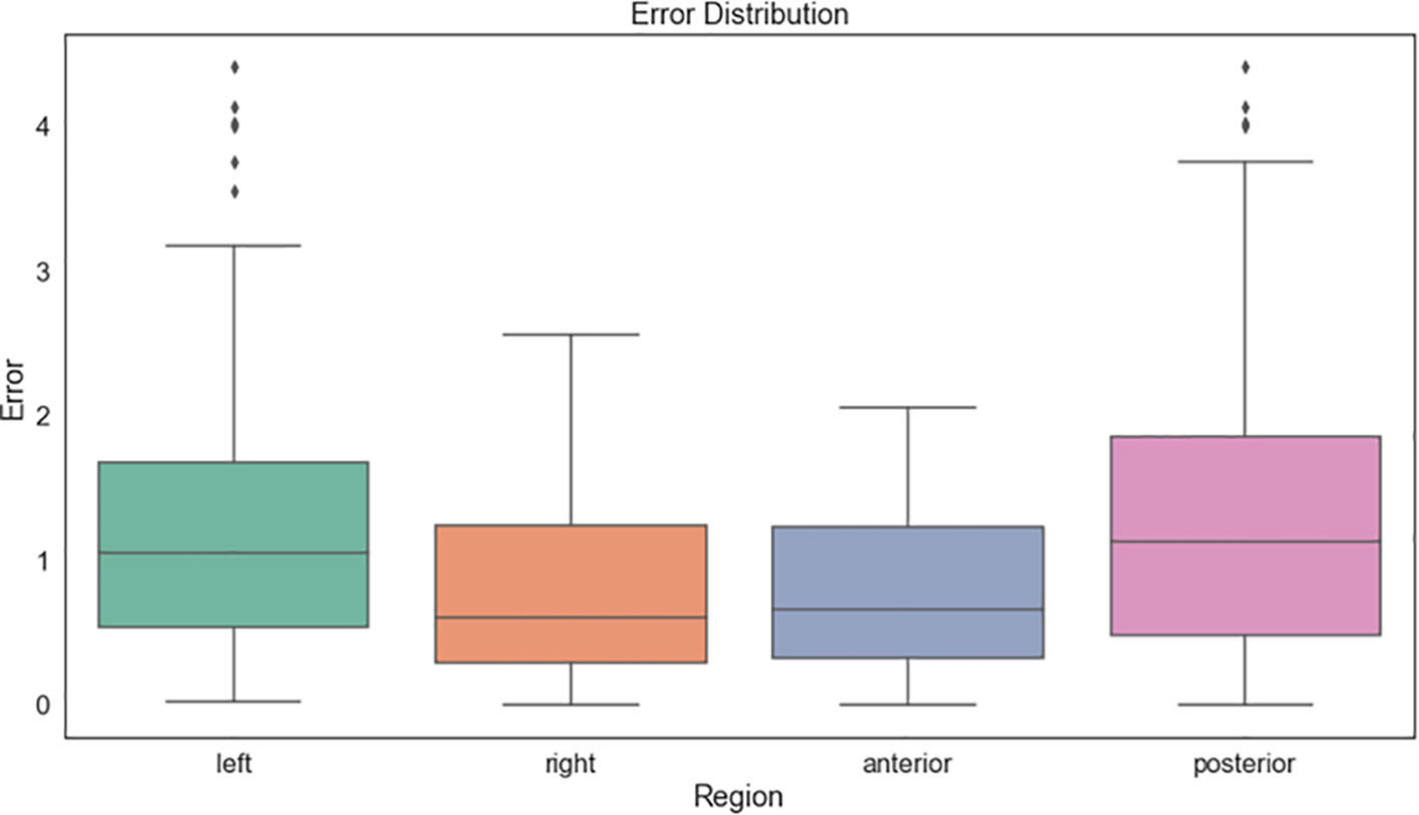
Distances between the resection margins collected intraoperatively with the navigation system and those identified in the postoperative CT.

The points collected along the surgical margins with the navigation system were compared with the real surgical margins identified in the postoperative CT by measuring their absolute distance. We separated the analysis into four regions divided by left and right sides and posterior and anterior locations. **Figure 5** displays the results.

**Figure 6:**
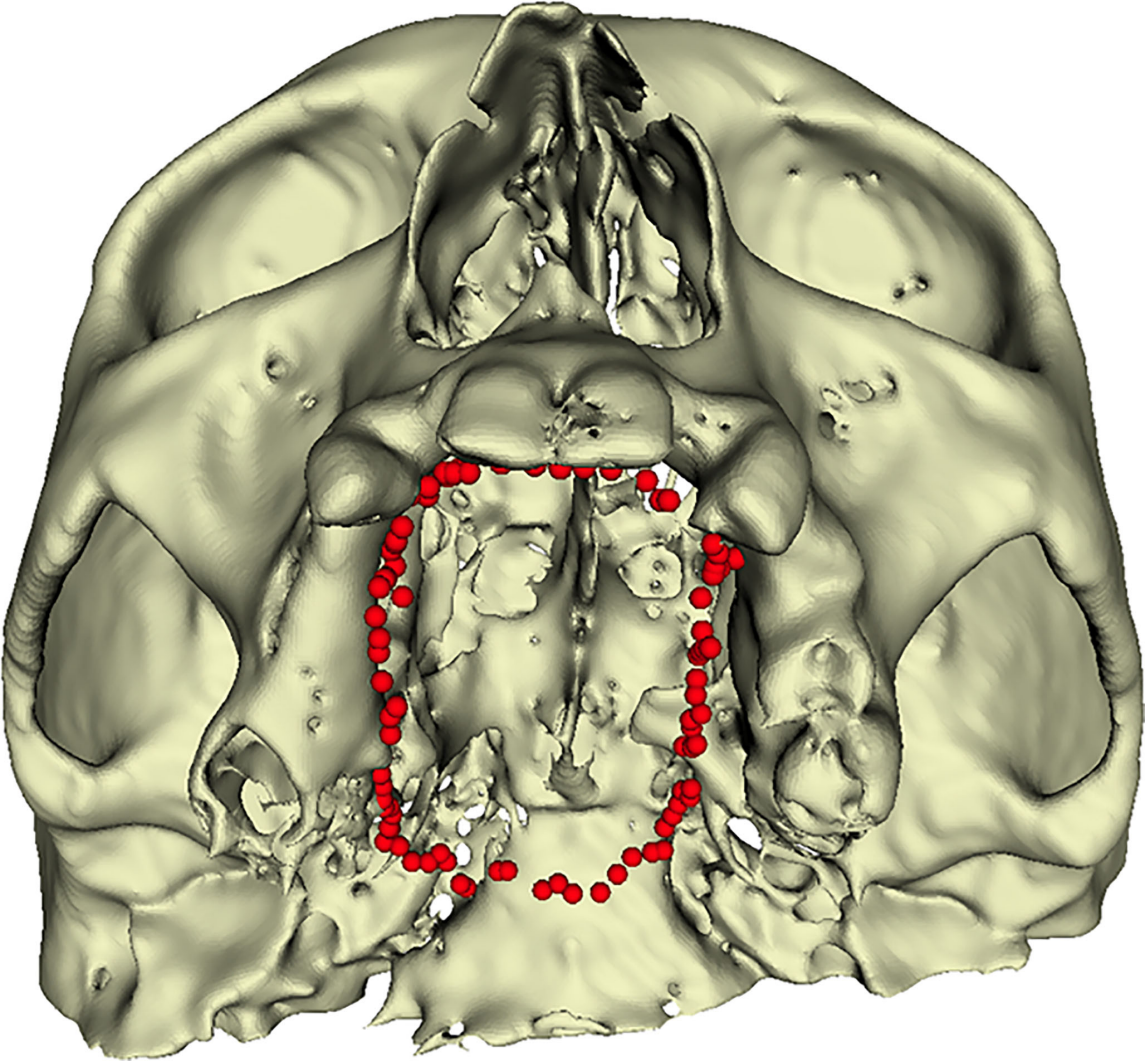
Points collected intraoperatively along the resection margins.

The mean distances were around 1 mm and below 2 mm in 90% of the samples. The posterior region presented higher errors compared to the anterior. The left side showed higher deviations than the right one. However, this deviation appears to be caused by the jig, which was still in place during the points collection and limited the pointer movements (**Figure 6**).

The AR app was also used inside the operating room, where surgeons visualized models of the patient’s anatomy overlayed on the camera’s image of an iPhone 6 (Apple Inc., Cupertino, CA, USA). The splint was inserted correctly and enabled AR display, where virtual models were represented aligned with the patient’s anatomy.

## Discussion

The resections of head and neck tumors in deep and less accessible regions represent complex surgical scenarios requiring extreme dexterity, as the field of view and instruments maneuverability is limited. For middle third tumors such as palate ACCs, the preservation of normal bony tissue and surrounding soft tissues should be maximized. Tel et al. (67) advocate an open “box resection” where the tumor is resected within a three-dimensional volume of healthy tissue. In these scenarios, CAS represents a valuable tool to plan (74), guide (9) and verify (49) the resection margins.

Pu et al. (75) concluded in their study with 37 patients that surgical margins can be predetermined without compromising oncological safety, and that the difficulty in determining these margins lies more on soft than hard tissue. Thus, the use of CAS should be limited to cases with mainly bone involvement (76). Ricotta et al. (74) demonstrated how performing a volumetric virtual plan of the resection can improve accuracy and reduce the probability of finding positive margins.

Intraoperative navigation is highly indicated for resections in areas with restricted access (8), where the deep portion of the tumor is not clearly visible. Such is the case of deep maxilla cuts, where Hasan et al. (63) have reported resections errors below 2 mm when using intraoperative navigation. Surgical navigation systems also helps in achieving R0 (absence of disease) in deep sinonasal tumors (77).

Midfacial tumors usually present a small soft tissue involvement with a predominant three-dimensional bone infiltration, making them adequate for virtual planning. Tumors may be located in areas with limited access, making surgical navigation a valuable tool in these scenarios. In addition, the proximity to vital structures that cannot be damaged adds another advantage for guided resection of centrofacial tumors.

Although the use of surgical guidance for tumor resections is a routine procedure in neurosurgery, the reports in the maxillofacial middle third tumors are scarce. The existing CAS applications mainly focus on virtual planning, intraoperative guidance for the free flap defect reconstruction, or validation of the reconstruction after trauma, not on the ablative procedure. Moreover, most existing commercial systems for surgical navigation use a three-point clamp (Mayfield clamp or similar) to fix the patient’s skull and prevent head movements. Then, a dynamic reference frame is attached to the clamp to define a reference system for the patient and perform the image-to-patient registration. This setup is suitable for neurosurgery, and it can help achieve high accuracy. However, in the resection of middle third tumors, surgeons need free movement of the head to adjust the line of sight with the surgical field and enable proper angulation of the saw and surgical tools. Therefore, other solutions for tracking and registration need to be found for these procedures.

Some studies have presented alternative setups for tracking and registration. Malham et al. (78) use the SpineMask (Stryker, Leibinger, Freiburg, Germany) to track the patient’s back and perform an automatic registration. This device is non-invasive, as it is an adhesive surgical tracker designed to be placed on the patient’s back. The device contains markers for automatic registration, which are placed surrounding the surgical field. Other studies remove the dynamic reference frame from their setup and use conventional optical cameras to detect fiducial markers and constantly update the registration. These setups are usually based on computer vision algorithms for the detection of adhesive skin markers (79) or anatomical features in the bone (21). Other frameless systems use different devices such as hyperspectral cameras to detect skin features (80) or advanced methods such as 3D digital image correlation (also called stereo DIC). This last solution presents precise real-time tracking at a lower cost and based on small markers. Xue et al. (81) tested it for tracking the maxilla after a Lefort I osteotomy. However, it was not tested in a clinical setup where light conditions and external factors such as blood or saliva can condition the tracking and accuracy of the system.

Most of the existing alternatives focus on anatomical regions presenting deformations, where installing a dynamic reference frame and performing a rigid registration becomes inaccurate. Others are designed for specific applications, such as SpineMask, and are not applicable for reduced regions like the mouth. Also, we consider the systems based on skin markers not adequate for an open transfacial approach. Registration markers must be placed surrounding the surgical field to ensure an accurate registration, but in our scenario, the space is limited and a rigid position between markers is difficult to maintain.

Tarsitano et al. (49) presented a surgical navigation setup for the resection of maxillary tumors in a study with twenty patients, obtaining promising results with clear margins in 91% of cases. In their setup, a dynamic reference frame was screwed to the patient’s skull. The registration was performed first with a point-to-point registration based on anatomical landmarks, obtaining a mean error of 2 mm, followed by surface matching for refinement. They computed the errors in preoperatively defined target points, finding values between 0.30 and 1 mm, and a mean error of 0.47 mm. Wei et al. (11) also used a similar setup in 15 patients with tumors involving the skull base, five of them presenting an ACC in the palate. In their case, they also installed the dynamic reference frame in the patient’s skull but used bony skull landmarks and tooth cusps for registration. The registration errors and the resection accuracy in this study were not reported. Although these studies present a feasible setup for ACC resection, the installation of the dynamic reference frame is invasive. Moreover, the use of anatomical landmarks and surface matching with points in the face presents a suboptimal registration for ACC resection. Points used for registration should be close to the surgical area and surround it to provide accurate results. Anatomical landmarks and tooth cusps are not clearly defined and subject to intra- and inter-observer variability, leading to higher errors.

In our study, we have explored two different configurations for tracking and registration in ACC resection. They provide a non-invasive installation of the reference frame allowing for head movements and a registration based on artificial landmarks located close to the surgical field. The dynamic reference frame is fixed to the patient’s teeth either through a silicone jig molded with the shape of the teeth or by means of a splint. The registration landmarks for the first solution (jig) consist of screws placed preoperatively. For the second solution, registration is performed through conical holes included in the splint. Both configurations were evaluated in an anatomical phantom providing equivalent accuracy results with no significant differences, and with deviations from the planned surgical margins of 0.57 and 0.61 mm respectively. These results are similar to the ones obtained by Tarsitano et al. (49). The jig and screws configuration was finally used in a surgical scenario of ACC resection, obtaining deviations from the surgical margin around 1 mm and below 2 mm in 90% of the collected points along the surgical margins. Errors were higher in deeper regions. This behavior was expected, since accuracy usually decreases in areas further from the registration landmarks (82, 83). The splint was also placed in the patient during the intervention for testing. Although the installation of screws in the maxilla has been previously described in other works as an unobtrusive and precise method for registration (68), we believe that using a splint could provide similar results while presenting a straightforward and less-invasive approach.

We have also proposed an alternative guiding method based on AR. A splint was again used for registration, to fix an AR marker in a known position and display virtual models of the patient. The precision of this system has been evaluated in a previous study, obtaining visualization errors below 3 mm (28). This solution was also evaluated in our phantom study, where we obtained a 0.4 mm (IQR = 0.89) median deviation from the planned surgical margins.

Previous studies have reported other registration methods for AR visualization. Gibby et al. (84) displayed the CT and virtual models indicating trajectories for pedicle screw placement in a lumbar spine phantom. They used the OpenSight software for the HoloLens to automatically register the data with the phantom. However, a manual adjustment was needed to correct the alignment. The pedicle screws were placed with deviations between 1.3 and 1.53 mm from their planned trajectory. Other studies rely only on manual alignment for registration (17) or use additional instruments like electromagnetic or optical tracking systems (19, 20).

Although all configurations are feasible, we found the AR app to be less convenient for this procedure, as the limited line of sight of the surgical field also restricts the movements and visibility with the smartphone. The sterilization of the smartphone was easily solved by introducing it in the sterile case. However, the need to hold the smartphone, leaving only one hand free, can present a limitation. The use of head-mounted displays such as HoloLens offers an alternative to the smartphone, although the possible points of view are as limited or more than with the phone. Therefore, AR can complement conventional navigation by allowing an inspection of the margins before or after surgery but is not adequate for resection guidance. The accuracy of the system depends on the quality with which the camera sees the AR marker. This factor is highly dependent on lighting conditions and the pose of the camera with respect to the marker. The detection is optimal when the camera is close to the marker and looks at it from the front. However, when the camera moves and detects the marker from a different angle, some inaccuracies can arise. This inherent holographic instability has already been noted by Gibby et al. (84) with the HoloLens.

## Conclusions

The resection of an ACC in the palate is very challenging due to the limited visibility and the proximity to vital structures. Surgical navigation becomes a valuable tool to ensure adequate margins in such complex scenarios while performing a conservative approach. This study proposes and evaluates three different navigation setups for ACC resection. All configurations aim to provide accuracy with a non-invasive surgical procedure, improving the solutions proposed in previous studies (11, 49). Apart from providing a less invasive solution, the novelty of the proposed setups relies on the fact that all configurations, including AR guidance, are based on 3D printing to fabricate tools that enable navigation of the patient and surgical instruments. The splint, dynamic reference frame, AR marker, and adaptor for tracking the surgical instrument are all 3D printed with desktop 3D printers at a low cost. The three solutions were evaluated in an anatomical phantom, where they provided similar results, and tested in a surgical case. The configuration using an optical tracker and screws for registration was chosen for resection guidance during the procedure.

Surgeons combined the transoral navigated surgery with a nasal endoscopic approach, performing an optimal resection while preserving the whole alveolar process of the maxilla and upper teeth. The postoperative CT scans showed adequate resection margins. The pathological result was low-grade adenoid cystic carcinoma cribriform type, invading the mucosa, hard palate, nasal septum, and nasal floor with clear margins and perineural invasion. Head and neck tumor board established surveillance without adjuvant radiotherapy and a close follow-up. After two years, the patient is free of disease.

The results obtained from this surgery showing the accuracy and convenience of the proposed setups are promising. Navigation provided the confidence needed to undertake a more conservative approach and avoided the complete removal of the maxilla. The proposed navigation setup allowed a less invasive procedure compared to previous studies. We believe that image-guided surgery and 3D printing can provide a personalized, safe, and conservative en-bloc resection minimizing the need for reconstruction.

## Data Availability Statement

The original contributions presented in the study are included in the article/**Supplementary Material**. Further inquiries can be directed to the corresponding author.

## Ethics Statement

The study was approved by the Research Ethics Committee at Hospital General Universitario Gregorio Marañón (Madrid, Spain) and performed in accordance with the principles of the 1964 Declaration of Helsinki as revised in 2013. The patient provided her written informed consent to participate in this study.

## Author Contributions

MG-S, RM-M, and DG-M designed the study and performed the data collection. MG-S wrote the manuscript and performed the analysis. GA contributed to the writing of the manuscript. SO and JP conceived the study. SO, CN-C and GS performed the surgery. All authors contributed to the article and approved the submitted version.

## Funding

This work has been supported by projects PI18/01625 (Ministerio de Ciencia, Innovación y Universidades, Instituto de Salud Carlos III and European Regional Development Fund “Una manera de hacer Europa”) and IND2018/TIC-9753 (Comunidad de Madrid).

## Conflict of Interest

The authors declare that the research was conducted in the absence of any commercial or financial relationships that could be construed as a potential conflict of interest.

## Publisher’s Note

All claims expressed in this article are solely those of the authors and do not necessarily represent those of their affiliated organizations, or those of the publisher, the editors and the reviewers. Any product that may be evaluated in this article, or claim that may be made by its manufacturer, is not guaranteed or endorsed by the publisher.
