# Supplementary material for: Surgical Navigation, Augmented Reality, and 3D Printing for Hard Palate Adenoid Cystic Carcinoma En-Bloc Resection: Case Report and Literature Review
Source: Front Oncol. 2022 Jan 4;11:741191. doi: 10.3389/fonc.2021.741191 (PMC8763795; doi:10.3389/fonc.2021.741191)
Supplement: Supplementary file 1 [file DataSheet_1.pdf]

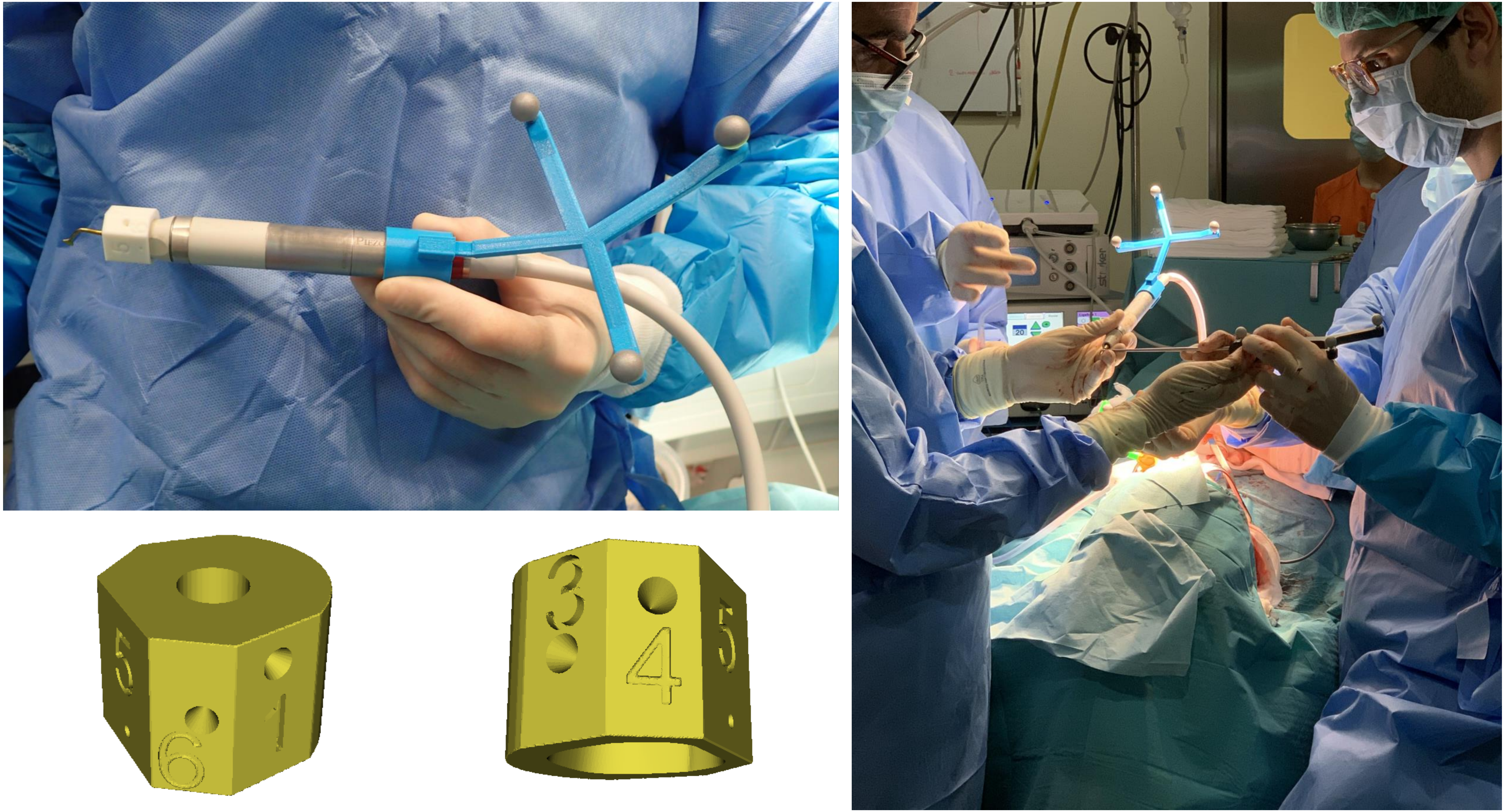

Supplementary Figure 1. Handpiece tracking and registration. Left pictures show the registration tool with conical holes (white in the top image, yellow at the bottom). The right image shows the step for the recording of the saw tip.
