# Supplementary figures and images for: Surgical Navigation, Augmented Reality, and 3D Printing for Hard Palate Adenoid Cystic Carcinoma En-Bloc Resection: Case Report and Literature Review
Source: Front Oncol. 2022 Jan 4;11:741191. doi: 10.3389/fonc.2021.741191 (PMC8763795; doi:10.3389/fonc.2021.741191)

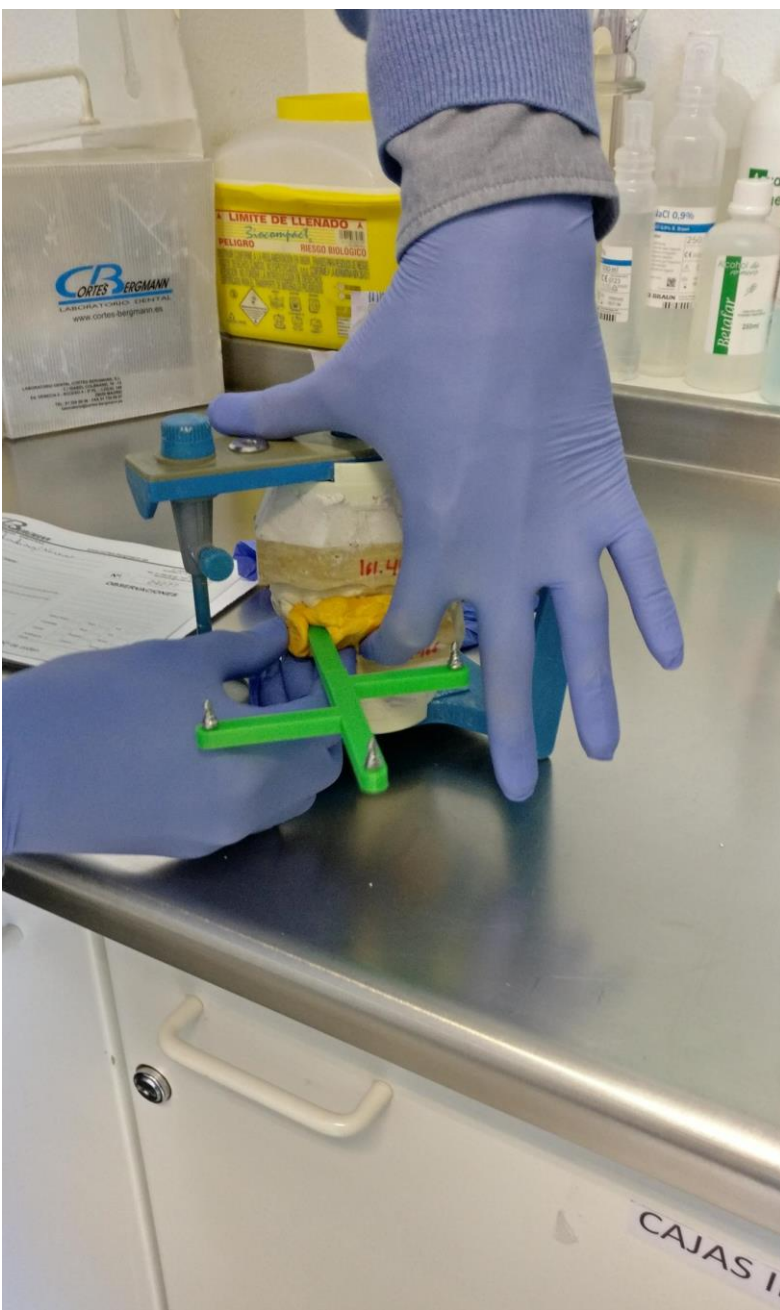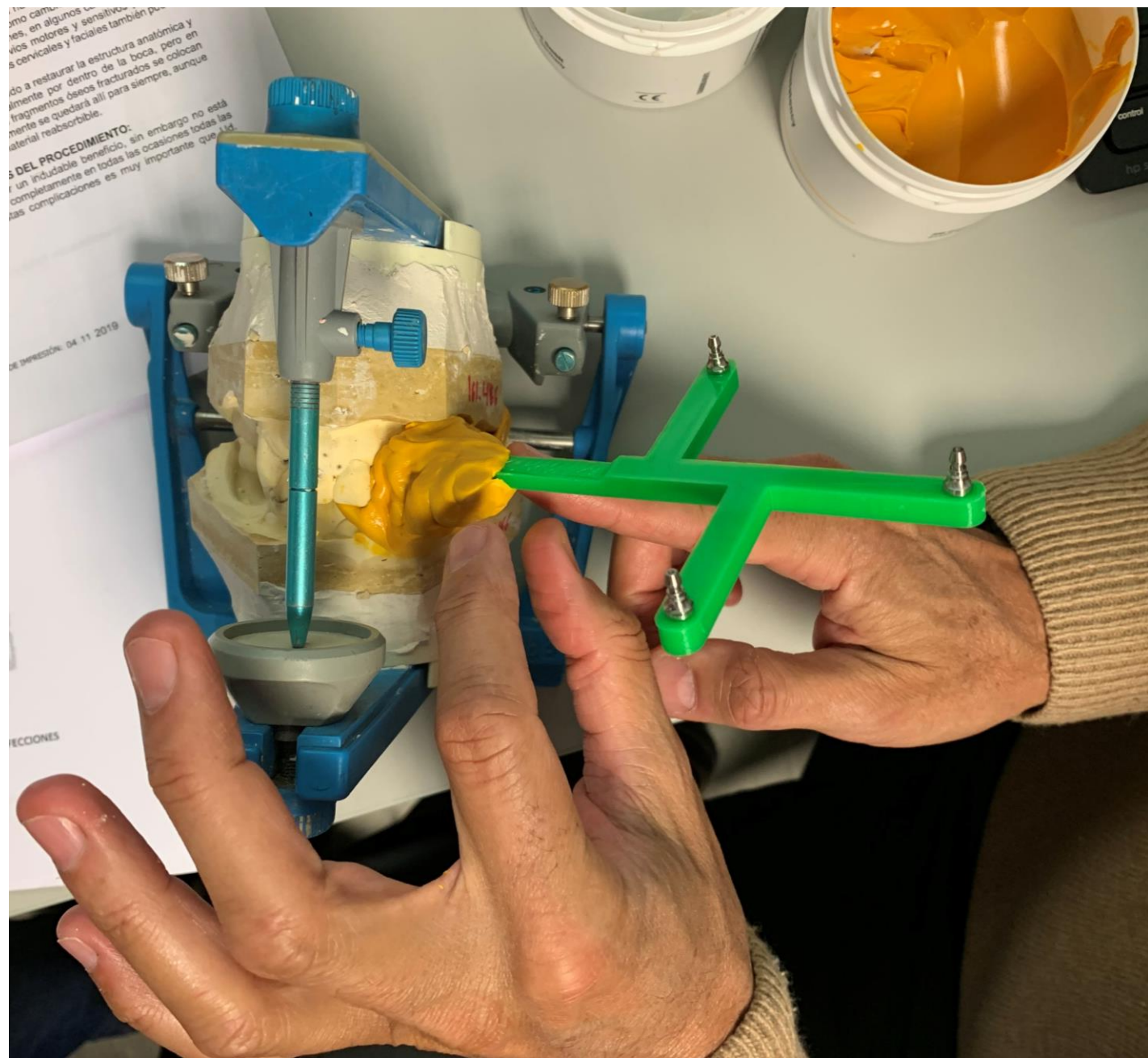

Supplementary Figure 2. Jig fabrication process.

Supplement: Supplementary file 2 [file DataSheet_2.pdf]
